# Effect of educational intervention on the knowledge, attitude and practice of breast self-examination among female students at a private university in Southern Nigeria

**DOI:** 10.1186/s12885-024-12116-w

**Published:** 2024-03-19

**Authors:** Rejoice Oritsemoyowa Uruntie, Chime Helen Oputa, Esegbue Peters, Agofure Otovwe

**Affiliations:** 1 Department of Public and Community Health, Novena University, Ogume, Delta State Nigeria; 2 Department of Public Health, Achievers University, Owo, Ondo State Nigeria

**Keywords:** Breast self-examination, Knowledge, Attitude, Practice, Female, Students, Nigeria

## Abstract

**Introduction:**

In Nigeria, breast cancer (BC), a disorder marked by the unchecked growth of breast cells, has been the commonest cancer among women in Nigeria. Breast self-examination (BSE) is one of the suggested methods for screening for the early diagnosis of breast cancer. However, studies have reported inadequate knowledge, negative attitudes and poor practices of BSE among undergraduate female students. The study was designed as an interventional study to examine the effect of educational intervention on knowledge, attitude and practice of breast self-examination among female students in a private university in southern Nigeria.

**Methods:**

This pretest posttest design study was carried out on 103 students of Novena University at baseline in 2022, which were chosen through the use of simple random sampling. A validated questionnaire with components on demographics, knowledge, attitude, and BSE practice was used to gather the data. After that, the students participated in three hourly sessions of an educational intervention for two weeks. A month later, the students’ data were once again collected, and SPSS 20 software was used to evaluate the results using the mean, paired t test, and logistic regression at the *P* < 0.05 level of significance.

**Results:**

The mean age of the respondents was 22.37 ± 1.92 years. Only 53 (51.3%) were aware of BSE. The mean knowledge, attitude and practice of BSE at pretest significantly increased at posttest after the educational intervention (1.58 ± 1.48 vs. 4.31 ± 1.15, 2.37 ± 1.27 vs. 4.80 ± 0.49 and 1.97 ± 0.09 vs. 5.81 ± 3.26, respectively). Furthermore, age and family history of BC were predictors of knowledge (OR = 4.00 95% CI = 0.29–41.99, OR = 141, 95% CI = 0.15–13.18), attitude (OR = 2.39, 95% CI = 0.28–12.32, OR = 1.15, 95% CI = 0.24–8.34) and practice of BSE (OR = 2.66, 95% CI = 0.38–18.41, OR = 1.44, 95% CI = 0.24–8.34) respectively.

**Conclusion:**

The findings showed that using an educational intervention strategy will improve the knowledge, attitude and practice of BSE among undergraduate students.

**Supplementary Information:**

The online version contains supplementary material available at 10.1186/s12885-024-12116-w.

## Background

The unchecked growth of breast cells is the hallmark of Breast Cancer (BC) [1]. In 2020, there were over 2 million cases of BC reported worldwide, making it the most common type of cancer [2]. Breast cancer usually starts in the breast tissue around the lobules that transport milk to the nipple [3]. Both men and women can develop BC [4], and it also affects women more frequently than any other type of cancer in both industrialized and developing nations [2].

Breast cancer is the most common type of cancer diagnosed and the second largest cause of cancer-related deaths in sub-Saharan Africa. According to Adewale [5], Sub-Saharan Africa has the highest age-standardised incidence rate globally, with 17.3 cases per 100,000 women annually. The regions of West Africa and Southern Africa, however, have the highest age-standardised incidence rates in sub-Saharan Africa, with 38.9 and 38.6, respectively, per 100,000 women annually [5, 6]. Based on country-specific prevalence, the most common African countries are Nigeria and Mauritius, with age-standardised incidences of 50.4 and 64.2 per 100,000 people, respectively [5, 6].

According to the Global Cancer Observatory [7], BC accounts for approximately 23% of all cancer diagnoses and 18% of cancer-related deaths in Nigeria, making it the country’s largest cause of cancer-related fatalities. Due to delayed presentation of the disease at medical facilities for treatment, BC is often diagnosed at an advanced stage, which raises the country’s high BC mortality rate [7, 8]. Surveys have shown that most Nigerian women, both in rural and urban areas, are unaware of the disease’s risk factors or symptoms [9, 10]. The risk factors of BC include sex, aging, estrogen, family history, gene mutations and unhealthy lifestyle [11].

Screening can identify BC early on, potentially saving lives [12]. In order to screen for BC, all women must be checked for the disease’s indicators, even if they don’t exhibit any symptoms. Early cancer detection is the aim of screening. Compared to later-stage malignancies, early-stage cancers have a higher chance of survival and are easier to treat. One’s risk of dying from breast cancer is reduced by routine screening [13]. Three tools are suggested for BC screening: mammography, clinical breast examinations (CBEs) by a doctor, and breast self-examinations (BSEs) [14]. Breast self-examination is a quick, easy, private, low-cost, non-invasive process that does not require any specific materials or tools. It may be applied in only five minutes and is an excellent way to diagnose BC [15].

The aim of this process is to familiarize women with their breasts’ appearance and feel, as well as to assist them in identifying any changes to their breasts as soon as feasible. Given that numerous studies have shown that university students do not practice BSE, training programs are crucial in promoting BSE and therapy among women [16]. For example, among female undergraduate students at the University of Buea in Cameroon, a study revealed that only 9.0% knew how to perform BSE, only 13.9% knew what to look for while performing BSE and only 3% had routinely completed BSE. Moreover, Nade et al. [17], stated that the primary deterrent to BSE performance was a lack of awareness about the procedure.

In a similar vein, data from another Nigerian study [18], revealed that only 19.0% of the participants were completing BSE consistently. Research by Birhane et al. [19], Salaudeen et al. [20], Isara and Oyedokun [21], Kayode et al. [22], Chioma et al. [23], Agboola et al. [24], Fidelis and Manalo, [25], and Bener et al. [26] further supports the low BSE behaviours among female university students.

As opposed to a screening technique, BSE practice is essential to increase awareness and transfer knowledge among populations at risk of developing breast cancer, according to the American Cancer Society [27]. Furthermore, women who accurately perform BSE once a month are more likely to notice a lump in its early stages, and early detection has been linked to earlier treatment and a higher chance of survival. Furthermore, intervention studies have documented the use of educational intervention in improving the awareness, knowledge, attitude and practice of BSE. A study in Akure South-West Nigeria documented the increase in the post-assessment awareness, attitude towards and practice of BSE after training [28]. Similarly, another study in Ibadan Oyo State also reported an increase in the post-assessment knowledge, attitude and practice of BSE among secondary school students after an educational intervention [29]. Other studies also documented post-assessment increase in knowledge, attitude and practice of BSE [30, 31]. There is currently no information available regarding how educational interventions affect female Novena University students’ knowledge, attitudes, and BSE practices.

**Fig. 1:**
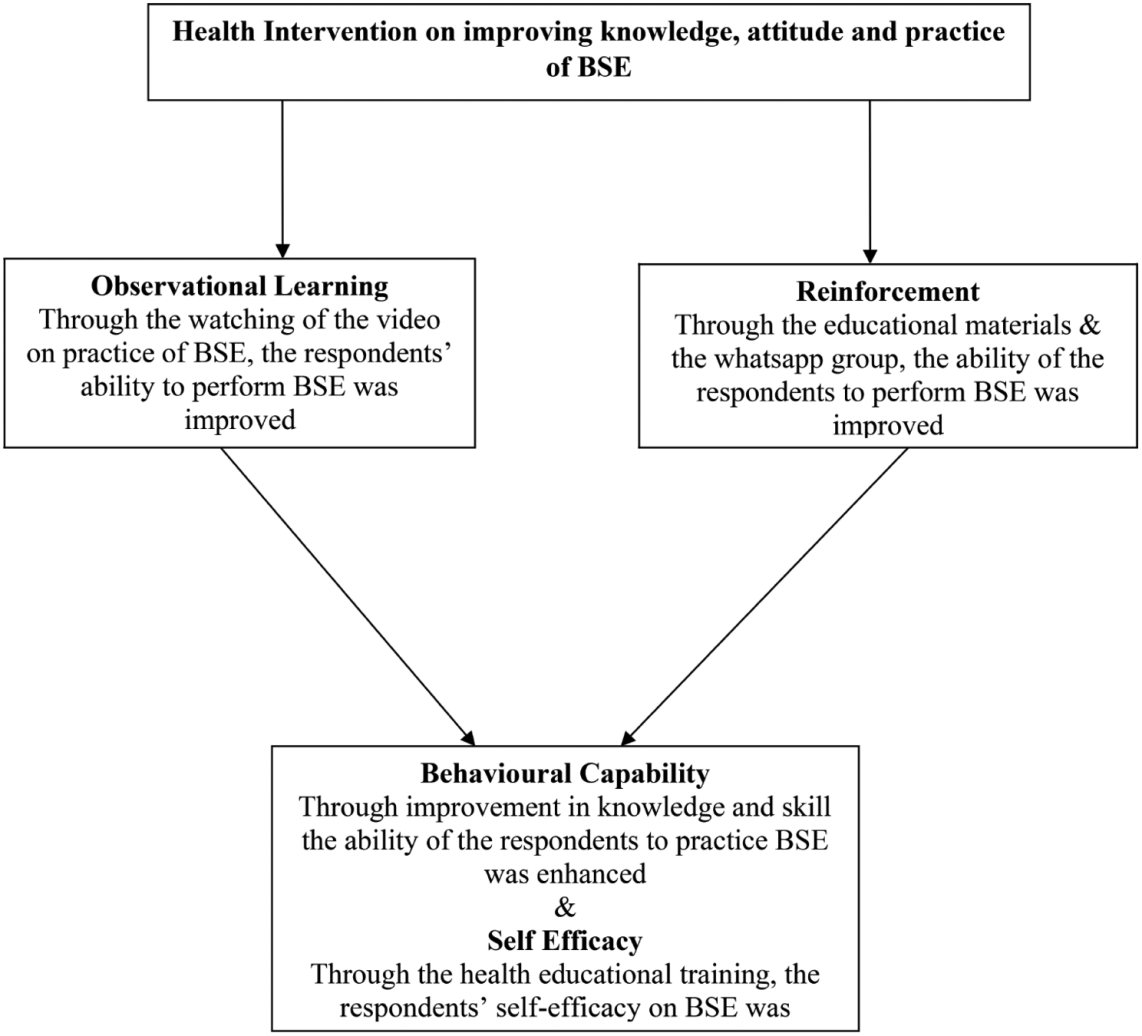
Using the social cognitive theory to explain the behavioural change among the respondents. *Source*: Adapted from [32]

To establish baseline data for future research as well as new curricular strategies about BSE in Nigerian universities generally, this study was designed to ascertain the effects of educational intervention on the knowledge, attitudes, and practices of female students regarding BSE at Novena University. Furthermore, as shown in Fig. 1 the social cognitive theory will be used to highlight the behavioural change in knowledge, attitude and practice of BSE among the respondents. The health intervention through observational learning, reinforcement is expected to strengthen the behavioural capability and self efficacy of the respondents to practice BSE.

## Methods

### Study design and settings

The study, which took place from January to June 2022 at Novena University in Ogume a rural area of Delta State, used a prepost quasiexperimental design to assess the effect of educational intervention on the knowledge, attitude, and practice of breast self-examination among female students. The checklist for the Transparent Reporting of Evaluations with Non-Randomized Designs (TREND) statement was used for reporting [33].

The research was conducted at Novena University, located in Ogume, a rural area of Delta State, Nigeria. Novena University was established in 2005 and is the first private institution in the state.

### Participants

The eligibility criteria for the study are that the selected undergraduate students must be female students of Novena University. They must be in their second and third year in their study. First-year students were excluded because they are freshers in the university system and might want to concentrate on their studies. Four hundred level students were excluded because they were in their exit classes and might not be available for follow-up data collection. Other exclusion criteria were as follows:

Female students in their second or third year of study in the chosen colleges and departments who were either pregnant or nursing at the time of the study were excluded from the study.

### Sample size estimation and sampling technique

The prepost quasiexperimental study formula for means was used to estimate the sample size [34].

$$\text{n}=\frac{(\text{Z}\alpha+\text{Z}\beta)^{2}\text{SD}^{2}}{\text{O}^{{\prime}{2}}}$$

where n = Minimum sample size.

Zα = 1.96 if *p* = 0.05 at 2 tailed.

Z_β_ = 0.84 if power = 80%.

SD = is the standard deviation (2.17) obtained from a previous study [35].

δ = µ_1_ and µ_2_ are the means before and after intervention, respectively, which are 13.55–14.24, which were obtained from a previous study [28].

$${\text{n}}\,{\text{=}}\,\frac{{{{\left( {1.96\,+\,0.84} \right)}^2}\, \times \,{{2.17}^2}}}{{{{\left( {0.69} \right)}^2}}}{\text{~}}$$

$${\text{n}}\,=\,77.54$$

A 20% nonresponse rate was added.

$$q\,=\,\frac{1}{{1 - f}}$$

where

$${\text{f}}\,=\,20\% \,=0.2$$

$$q\,=\,\frac{1}{{1 - 0.2}}$$

$$q\,=\,\frac{1}{{0.8}}$$

$$q\,=\,1.25$$

$${\text{n}}\,=\,77.54\times \, 1.25$$

$${\text{n}}\,=\,96.93$$

$${\text{n}}\,=\,97$$

Nonetheless, at the study’s baseline, 103 students were sampled.

The study utilised a stratified random sampling technique. The College of Management and Social Sciences, the College of Natural and Applied Sciences, and the College of Medical and Health Sciences comprised the entire university. The three colleges were stratified into 18 departments. Out of the 18 departments, 10 departments were randomly selected. The estimated female students to be sampled from each department were performed using proportionate sampling shown in Table 1. From each department, a subset of eligible female students was then chosen using a simple random selection procedure.

**Table 1:** Sociodemographic characteristics

Variable	**Frequency **(*N* = 103)	Percentage
**Age (Years)**		
16–20	45	43.6
21–25	35	34.0
26–30	18	17.5
31–35	5	4.9
**Religion**		
Christian	102	99.0
Islam/Muslim	1	1.0
**Marital Status**		
Single	94	91.3
Married	9	8.7
**Level**		
200	57	55.3
300	46	44.7
**Have you heard about breast cancer**		
Yes	102	99.0
No	1	1.0
**Do you have any family history of breast cancer**		
Yes	6	5.8
No	97	94.2
**If yes, who in your family**		
Grandmother	3	42.9
Aunty	4	57.1
**Have you heard about breast self-examination**		
Yes	53	51.5
No	50	48.5
*If yes, what is your source of information		
Television	28	13.8
Radio	17	8.4
Books	28	13.8
Friends	26	12.8
Internet	39	19.2
Health worker	41	15.3
School	34	16.7
**Father’s educational level**		
None	5	4.9
Primary	5	4.9
Secondary	26	25.2
Tertiary	67	65.0
**Mother’s educational level**		
None	5	4.9
Primary	10	9.7
Secondary	43	41.7
Tertiary	45	43.7
**Department**		
Accounting and Finance	22	21.4
Mass Communication	15	14.6
Nursing	25	24.3
Public and Community Health	26	25.2
Medical Laboratory Sciences	2	1.9
Political Science	2	1.9
Optometry	5	4.9
Intelligence and Security Studies	1	1.0
Sociology	2	1.9
Pharmacology	3	2.9

Mean age: 22.37 ± 1.92

*Analysed as multiple response

### Instrument of data collection

A self-administered validated questionnaire served as the data gathering tool. The instrument was pretested among 10% of the sample size in the university. The researchers ensured that the students used for pretest were not included in the final study. It is made up of closed-ended, open-ended, and precategorized questions that were created following a thorough analysis of pertinent literature demonstrating the effect of educational intervention on female students’ knowledge, attitudes, and practices regarding BSE. Four sections make up the questionnaire, numbered A through D. Section A focused on the sociodemographic characteristics of the students (such as age, marital status, religion, department of study, and level of study).

Section B focused on students’ knowledge of breast self-examination, Section C on their attitude toward breast self-examination, and Section D evaluated their practice of breast self-examination.

### Implementation of the intervention

The training program was created based on the knowledge gaps that were found in the baseline data, a review of the literature, the WHO-recommended training manual, which included videos for breast self-examination (BSE), and other educational resources that were readily available. A PowerPoint presentation with the training guide’s material for each training session was used to provide the intervention’s topics. As a teaching tool, printed educational materials with a focus on BSE were employed. The researcher’s educational intervention was a group-based instructional training session. The program for health education intervention comprises a variety of activities, such as group discussions, lectures, and sessions. The training took place on the school’s premises on April 16 and 23, 2022. Saturdays were chosen for the training because that was the day the students had off from lectures. The researcher instructed the students for two weeks between April 16 and 23, 2022, in three hourly sessions. The study participants received the created curriculum as follow-up materials. The training guide covers the anatomy of the breast and common problems that women face with it. It also provides an overview of important topics related to breast self-examination, emphasizing its definition, common misconceptions, significance, application, technique, and rules. Finally, it includes a practical demonstration of how, when, and what to look out for during BSE, along with group work aimed at enhancing BSE knowledge, attitude, and practice. A video was included to provide a better picture of the step-by-step process of BSE performance. In addition, following each training session, the participants were free to ask questions.

### Outcomes

The primary finding of the research was the difference in the students’ pre- and posttest mean BSE knowledge, attitudes, and practices.

### Method of data collection

Two stages of the questionnaire development were utilized to evaluate the students’ BSE knowledge, attitudes, and practices. A group of students completed a self-administered questionnaire as part of a pretest evaluation of their knowledge, attitudes, and BSE practices at baseline. After that, utilizing the created training guide, the researcher ran a two-week BSE intervention session with the students every three hours. The students’ posttest results were gathered following a month-long follow-up. A WhatsApp group was created for the study participants to guarantee proper follow-up and minimize attrition.

### Data analysis

Using the Statistical Package for Social Sciences (SPSS) version 20.0, the pre- and posttest questionnaires were gathered, cleaned, manually coded, and then input into the computer. Frequency tables, charts, and mean knowledge, perception, and attitude ratings about DM prevention were created using descriptive statistics. To display the mean scores and standard deviations at the pre- and posttest, paired sample t test statistics were employed. Paired differences, the t-statistic, confidence intervals (95% CI), and the significant value at *P* < 0.05 were all supplied along with the mean differences between the pre- and posttest scores.

### Scales of measurement

#### Knowledge scale

The respondents’ level of BSE knowledge was measured using a dichotomous knowledge scale that was created. Six test items made up the knowledge component of the questionnaire. One point was awarded for a correct response, and zero was awarded for a bad response. As a result, the knowledge scores of each research participant were coded as > 3 and between 0 and 3. Responses with a score of 0–3 (Code 1) were considered to have poor BSE knowledge, while those with a score of > 3 (Code 2) were considered to have good BSE knowledge.

#### Attitude scale

A binary scale (agree, disagree) was developed to measure attitude. The attitude section contained five test items in total. One was assigned to a correct response, and zero was assigned to a wrong response. Consequently, the attitude scores of each research participant were classified as between 0 and 2 as Code 1 and > 2 as Code 2. Respondents with scores of > 2 = Code 2 were deemed to have a positive attitude toward BSE, whereas those with scores between 0 and 2 = Code 1 were considered to have had a negative attitude toward BSE.

#### Practice scale

The practice of BSE among the respondents was measured using a practice scale that was created. In the practice phase of the questionnaire, there were nine test items in total. One point was awarded for a correct response, and zero was awarded for a wrong response. As a result, the practice scores of each research participant were classified as falling between 0 and 4 (Code 1) and > 4 (Code 2). Respondents with a score of > 4 = Code 2 were deemed to have good BSE practice, whereas those with a score of 0–4 = Code 1 were considered to have demonstrated poor BSE practice.

## Results

Table 1 shows that 45 (43.7%) were between the ages of 16–20 years, while the majority were Christians 102 (91.3%), and 94 (91.3%) were singles. In addition, the majority of the respondents (102, 99.0%) were aware of breast cancer, with a few (6, 5.8%) having a family history of breast cancer. Additionally, about half of the respondents (53, 51.5%) had heard of BSE. The respondents’ source of information was health workers (41, 15.3%), internet (39, 19.2%), school (34, 16.7%) and television (28, 13.8%).

In Table 2, since only 53 (51.5%) of the respondents affirmed having heard of BSE at baseline, further analysis at baseline was subjected to only these respondents. Approximately 15 (28.3%) defined BSE as a way a person can check their own breast by feeling for lumps or other changes, and 8 (15.1%) defined BSE as a process whereby a person went for checkup to know if there was lumps in the breast. At posttest, approximately 27 (33.3%) defined BSE as the practice of examining one’s own breast 5–7 days after menstruation and 21 (25.9%) as a female looking at her breast to see if there is a change in size or reduction. Furthermore, at baseline, 13 (24.5%) affirmed that there are 3 steps involved in BSE, and 10 (18.9%) affirmed 5 steps. At posttest, 45 (55.6%) affirmed 5 steps, and 25 (30.9%) affirmed 4 steps.

**Table 2:** Knowledge of breast self-examination

Variable	Pre test		Post test	
	**Frequency **(*N* = 53)	Percentage	**Frequency **(*N* = 81)	Percentage
**What then is breast self-examination in your own understanding**				
I do not know	2	3.8	0	0
The process of checking the breast to know the current state, some signs and symptoms affecting it	4	7.5	13	16.0
The practice of examining one’s own breast 5-7days after menstruation	2	3.8	27	33.3
A step by step method women can use to examine their breast by looking at and feeling their breast regularly	6	11.3	4	4.9
Examining your breast yourself to check if there is any change	6	11.3	0	0
A female looking at her breast to see if there is a change in size or reduction	1	1.9	21	25.9
A way a person can check their own breast by feeling for lumps or other changes	15	28.3	1	1.2
Ability to educate yourself on breast maintenance and development which reduces breast cancer in women	4	7.5	7	8.6
A process whereby a person went for checkup to know if there is lumps in the breast	8	15.1	5	6.2
Self-examination of breast by mere touching	2	3.8	1	1.2
Physical inspection of the breast to detect early signs of breast cancer	3	5.7	2	2.1
**How many steps are involved in breast self-examination**				
I don’t know	16	30.2	1	1.2
2	4	7.5	1	1.2
3	13	24.5	8	9.9
4	9	17.0	25	30.9
5	10	18.9	45	55.6
7	1	1.9	1	1.2
**Have you had any breast problems before**				
Yes	4	7.5	8	9.9
No	49	92.5	73	90.1
**Do you know the incident rate of breast cancer in Nigeria**				
Yes	8	15.1	25	30.9
No	45	84.9	56	69.1
*Who should practice breast self-examination				
Pregnant women	12	8.5	9	3.4
Women with family history of cancer	21	14.9	48	18.1
Women with breast problems	16	11.3	51	19.2
Women with symptoms of cancer	19	13.5	50	18.9
Sexually active women	13	9.2	49	18.5
Non pregnant women	13	9.2	17	6.4
All women	47	33.3	41	15.5
**The best time for breast self-examination is**				
A particular day of each month	16	30.2	4	4.9
Within five to seven days after menstruation	22	41.5	64	79.0
Anytime I remember	15	28.3	13	16.0
**Breast self-examination should be done**				
Daily	8	15.1	3	3.7
Weekly	8	15.1	1	1.2
Monthly	29	54.7	60	74.1
Yearly	1	1.9	1	1.2
Not sure	7	13.2	16	19.8

*Analysed as multiple response

In Table 3, at pretest, the majority of the respondents (49, 92.5%) agreed that breast self-examination can reduce cancer death in women, while at posttest, all respondents (81, 100%) agreed that breast self-examination can reduce cancer death in women. In addition, at pretest, only 19 (35.8%) erroneously agreed that breast self-examination is a cure for breast cancer and other breast problems, while at posttest, that number decreased to 12 (14.8%).

**Table 3:** Attitude towards breast self-examination

Variables	Pre test		Post test	
	**Frequency **(*N* = 53)	Percentage	**Frequency **(*N* = 81)	Percentage
**I believe breast self-examination can reduce cancer death in women**				
Agree	49	92.5	81	100
Disagree	4	7.5	0	0
**I believe breast self-examination is important for the early detection and prevention of breast cancer and other breast problems**				
Agree	52	98.1	81	100
Disagree	1	1.9	0	0
**Do you think breast self-examination is a cure to breast cancer and other breast problems**				
Agree	19	35.8	12	14.8
Disagree	34	64.2	69	85.2
**I believe the practice of breast self-examination is a sin**				
Agree	1	1.9	4	4.9
Disagree	52	98.1	74	95.1
**I believe there is cure or long term survival rate for breast cancer if detected early**				
Agree	48	90.6	81	100
Disagree	5	9.4	0	0

In Table 4, at pretest, 33 (62.3%) of the respondents affirmed practicing BSE, while the majority (30.9%) affirmed starting practicing BSE at ages 15–24 years. At posttest, 53 (65.4%) affirmed practicing BSE, while 35 (66.0%) affirmed starting practising BSE at ages 15–24 years. Out of those that practice BSE at pretest, 9 (27.3%) affirmed applying four and five steps, while at posttest, 31 (58.5%) affirmed applying five steps and 18 (34.0%) four steps.

**Table 4:** Practice of breast self-examination

Variables	Pre test		Post test	
	**Frequency **(*N* = 53)	Percentage	**Frequency **(*N* = 81)	Percentage
**Do you practice breast self-examination**				
Yes	33	62.3	53	65.4
No	20	37.7	28	34.6
**If yes, how often do you practice breast self-examination**				
Once a month	8	24.2	44	83.0
Anytime I feel pain on the breast	8	24.2	7	13.2
Anytime I remember	17	51.5	2	3.8
**At what age did you start practicing breast self-examination (in years)**				
15–24	30	90.9	35	66.0
25–34	3	9.1	18	34.0
**Has a Doctor ever examined your breast**				
Yes	8	15.1	0	0
No	45	84.9	81	100
**If you do not practice breast self-examination, what are your reasons**				
I do not know how to do it/lack of knowledge	11	50.0	5	17.9
It is not necessary	9	40.9	3	10.7
I believe that I cannot be a victim of breast cancer	2	9.1	5	17.9
I forgot	0	0	15	53.6
**How many steps do you apply during breast self-examination**				
Seven	2	6.1	1	1.9
Five	7	21.2	31	58.5
Four	9	27.3	18	34.0
Three	9	27.3	3	5.7
Two	3	9.1	0	0
I do not know	3	9.1	0	0
*What do you look out for during breast self-examination				
Lumps	32	20.8	53	14.4
Swelling	22	14.3	53	14.4
Redness	19	12.3	52	14.1
Discharge	21	13.6	53	14.4
Change/difference in breast size	24	15.6	53	14.4
Wound	11	7.1	52	14.1
Pain	25	16.2	53	14.4

* Analysed as multiple response

In Table 5, the mean scores of knowledge increased from 1.58 ± 1.48 at pretest to 4.31 ± 1.15 at posttest. Additionally, the attitude scores increased from 2.37 ± 1.27 at pretest to 4.80 ± 0.49 at posttest.

**Table 5:** Prepost test scores of the outcome variables

Variable	Pretest	Posttest	t test	P value	95% CI	
					Lower	Upper
Knowledge	1.58 ± 1.48	4.31 ± 1.15	−0.715	0.030	0.2419	0.5136
Attitude	2.37 ± 1.27	4.80 ± 0.49	2.061	0.043	0.0178	1.0192
Practice	1.97 ± 0.09	5.81 ± 3.26	−5.734	0.000	−4.6398	−2.2491

**Fig. 2:**
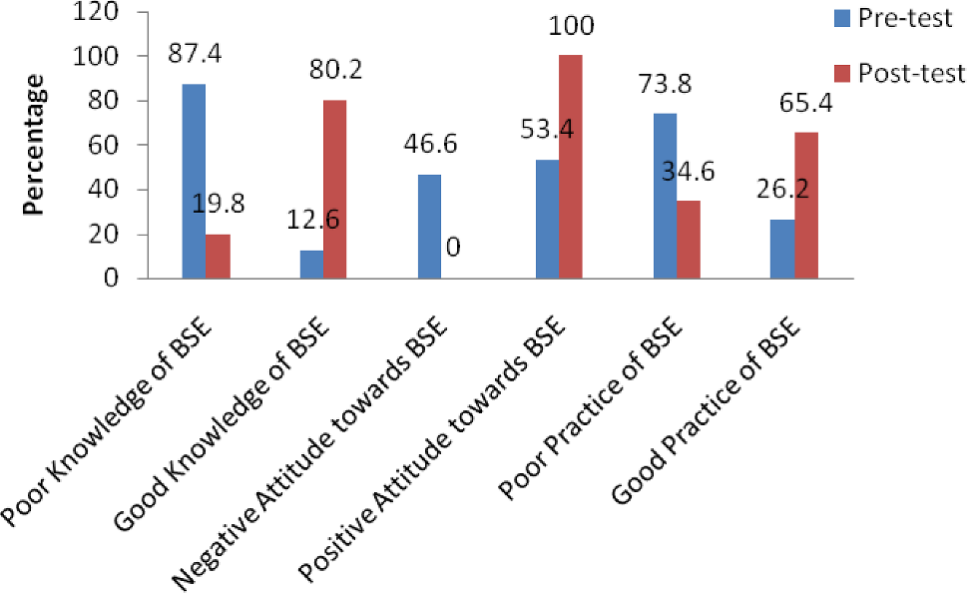
Pre- and posttest knowledge, attitudes and practices regarding BSE among the respondents

In Fig. 2, the posttest good knowledge of BSE increased from 12.6% at pretest to 80.2% at posttest. Additionally, the level of good BSE practices of the respondents increased from 26.2% at pretest to 65.4% at posttest.

In Table 6, respondents aged 31–35 years were more likely to be knowledgeable about BSE (OR = 4.00). 95% CI = 0.29–41.99), exhibited a positive attitude towards BSE (OR = 2.39, 95% CI = 0.28–12.32) and practice of BSE (OR = 2.66, 95% CI = 0.38–18.41). In addition, respondents who were single were more likely to be knowledgeable about BSE (OR = 1.17, 95% CI = 0.13–10.20) and exhibited a positive attitude towards BSE (OR = 2.47, 95% CI = 0.58–10.49) and good BSE practices (OR = 3.05, 95% CI = 0.36–25.67). Furthermore, respondents with a family history of breast cancer were more likely to be knowledgeable about BSE (OR = 1.41, 95% CI = 0.15–13.18), exhibited a positive attitude towards BSE (OR = 1.15, 95% CI = 0.16–4.50) and had good BSE practices (OR = 1.44, 95% CI = 0.24–8.34).

**Table 6:** Logistic regressional analysis of the factors predicting knowledge, attitudes and practices regarding BSE

	Knowledge				Attitude				Practice			
	Sig	OR	95% CI		Sig	OR	95% CI		Sig	OR	95% CI	
			L	U			L	U			L	U
**Age**												
16–20 (r)												
21–25	0.27	2.33	0.51	10.52	0.06	1.25	0.96	5.96	0.37	1.60	0.56	4.50
26–30	0.09	3.50	0.79	20.10	0.69	1.87	0.41	3.73	0.26	2.00	0.58	6.79
31–35	0.32	4.00	0.29	41.99	0.51	2.39	0.28	12.32	0.32	2.66	0.38	18.41
**Marital status**												
Single	0.88	1.17	0.13	10.20	0.21	2.47	0.58	10.49	0.30	3.05	0.36	25.67
Married (r)												
**Level**												
200 (r)												
300	0.19	2.19	0.66	7.21	0.57	1.26	0.57	2.74	0.18	1.82	0.74	4.40
**Family history of BC**												
Yes	0.76	1.41	0.15	13.1	0.86	1.15	0.16	4.50	0.68	1.44	0.24	8.34
No (r)				8								
**Awareness of BSE**												
Yes	0.97	5.12	0.16	3.65	0.00	1.24	0.09	1.41	0.99	1.61	0.45	2.41
No (r)												

## Discussion

The aim of the study was to determine the effect of an educational intervention on the knowledge, attitudes and practices of BSE among female students in Novena University Ogume Delta State, Nigeria.

The study’s conclusion demonstrated that following the BSE educational intervention, respondents’ knowledge, attitude, and practice scores increased in terms of both proportion and mean. According to the World Health Organization, [36], BSE is one of the screening techniques in which a woman examines her breast to learn about its topography to spot any abnormal lumps, distortions, discharges, or swellings with the goal of detecting them early for quick treatment initiation and improved survival rates for BC patients. As per this definition, some of the respondents at baseline were able to define BSE with the proportion of those who were able to define BSE increasing at postintervention. Knowledge of the steps of BSE among the respondents shows that while some of the respondents affirmed not knowing the steps involved in BSE at baseline, the proportion of the respondents who affirmed knowing the steps involved in BSE increased at postintervention. The BSE educational intervention may be responsible for the observed rise in knowledge. In the same vein, the respondents’ general level of BSE knowledge increased after the intervention. The results were in accordance with a study conducted in Bangladesh, where female students showed poor baseline BSE knowledge but markedly improved postintervention [37]. The findings were also in line with those of other studies in Egypt [38], Iran [39] and Turkey [40]. The results aligned with the outcomes of prior research conducted in Nigeria on students examining the impact of educational interventions on enhancing BSE knowledge [28, 41]. The implication of this finding is that health educational intervention is a reliable strategy to improve the awareness and knowledge of BSE among undergraduate students.

In a similar vein, all of the respondents showed a positive attitude about BSE, indicating a significant boost in their attitudes toward BSE after the intervention. The majority agreed that while BSE is not a treatment for breast cancer, it is crucial for the early diagnosis and prevention of the disease. Additionally, this was in line with the results of a prior study conducted in Iran and Nigeria, which found that postintervention attitudes concerning BSE and breast cancer screening had improved [28, 39]. The health education intervention may have contributed to the attitudinal increase that was seen at the postintervention. This underlines the importance of health education as a strategy for improving attitudes towards BSE among students.

Similarly, compared to the baseline, the respondents’ mean and proportional practice scores increased after the intervention. The percentage of respondents stating that they did not know how to practice BSE dropped, while the percentage stating that they were aware of the processes involved in practising BSE rose. It should be highlighted, nonetheless, that even though BSE practice has increased, some study participants forgot to complete a BSE practice assignment that was provided to them during the training. This was in spite of repeated prompts sent through the established WhatsApp group. This was comparable to other research reports [42–44] in which study participants mentioned forgetfulness as one of the reasons they did not practice BSE. This demonstrates that to bring about the intended shift in behaviour towards BSE practices, ongoing health education combined with reinforcement is needed. This result was in line with other research conducted in Nigeria [28, 41], Bangladesh [37], and Iran [45].

Older age was correlated with BSE knowledge, attitudes, and practices. Compared to younger responders, those between the ages of 21 and 35 were more likely to practice BSE. This may be explained by the fact that older respondents belonged to higher social strata and hence may have had more exposure to the risks associated with BC. This study had similarities to another conducted in Ethiopia, where a higher age was found to be a predictor of BSE practice [46]. Corroborating this finding was the fact that respondents at a higher level of study (300 level) were more likely to be knowledgeable about BSE and exhibit a positive attitude and practice of BSE. This shows that the focus of BSE intervention programmes should focus on younger students who might not be well informed and knowledgeable about BSE. This may contribute to both a rise in BSE practice and a decrease in the probability of early breast cancer identification, which is critical for breast cancer treatment. A similar study conducted in Ghana among female secondary and tertiary school students revealed that older tertiary school students had greater BSE knowledge and practice [47]. Similarly, respondents who were single were more likely to be knowledgeable about BSE and exhibit a positive attitude and practice of BSE. This was in consonance with previous studies in Nigeria and Turkey were marital status was significantly associated with knowledge and practice of BSE [48, 49]. This observation could be explained by the fact that the single respondents were more in the current study and may be more conscious of their health than married respondents and so might be more likely to be knowledgeable and practice BSE than married respondents. This result, however, was at odds with those of a study conducted among Ethiopian women, which found that married, widowed, and divorced study participants knew more about and used BSE than single study participants [50]. The fact that the Ethiopian study included older women between the ages of 20 and 79 in its sample may account for the differences in the research results.

Furthermore, those who had a family history of BC were more likely to be informed, have a good outlook, and practice BSE. It is possible that a family member’s history of cancer will inspire them to prevent the disease. Previous research conducted in different African nations supported this conclusion [50–52]. Additionally, there was a higher likelihood of knowing and practicing BSE among respondents who were aware of BSE. Knowledge that has been demonstrated to increase the likelihood of practicing BSE can be motivated by awareness [46].

The limitation of the study stems from the fact that the study relied solely on self-reported responses especially after the intervention which could be subject to recall bias. However, the authors ensured that the students were given enough time when filling the questionnaire and they were allowed to ask questions for clarity whenever the need arises.

## Conclusion

The study shows that using an educational intervention strategy will improve the knowledge of BSE, attitude towards BSE and practice of BSE among undergraduate students. It was therefore recommended that this strategy be adopted by the government, nongovernmental organizations involved in cancer research and the management of higher institutions in Nigeria to improve the rate of early detection and treatment of breast cancer, which in the future could lower the disease’s morbidity and death rate, particularly for young women.

### Electronic supplementary material

Below is the link to the electronic supplementary material.

Supplementary Material 1

## Data Availability

The datasets analyzed during the current study will be available from the corresponding author on reasonable request.

## Abbreviations

BC: Breast Cancer
BSE: Breast Self-Examination
OR: Odds Ratio
CI: Confidence
CBEs: Clinical Breast Examinations
WHO: World Health Organisation
Sig: Significance
