# Correction: Effect of educational intervention on the knowledge, attitude and practice of breast self-examination among female students at a private university in Southern Nigeria

**DOI:** 10.1186/s12885-024-12152-6

**Published:** 2024-03-25

**Authors:** Rejoice Oritsemoyowa Uruntie, Chime Helen Oputa, Esegbue Peters, Agofure Otovwe

**Affiliations:** 1 Department of Public and Community Health, Novena University, Ogume, Delta State Nigeria; 2 Department of Public Health, Achievers University, Owo, Ondo State Nigeria

**Correction: BMC Cancer 24, 355 (2024)**

10.1186/s12885-024-12116-w

Following publication of the original article [1], the authors noticed a typesetting error, whereby the first author, Rejoice Oritsemoyowa Uruntie, was erroneously omitted from the author group. The publishers apologise for this error and any inconvenience caused.

The original article [1] has been corrected.

## References

[1] Uruntie RO, Oputa CH, Peters E, Otovwe A (2024). Effect of educational intervention on the knowledge, attitude and practice of breast self-examination among female students at a private university in Southern Nigeria. BMC Cancer.

